# The Child is Father to the Man

**DOI:** 10.1007/s11606-026-10510-7

**Published:** 2026-05-12

**Authors:** Lealani Mae Y. Acosta

**Affiliations:** Department of Neurology, Vanderbilt Health, Nashville, TN USA

I grasp her hands as she steadies mine

the cherry red curlicue of the stovetop

shiny green pills that rattle deliciously in a bottle

Mother’s purse contents upended

no, no, no, don’t touch

Oreo cookie sleeves vanish from the cupboard

like a magician’s scarves

disappear into bedsheets, sock drawers, ample cheeks

no, no, no, don’t touch

the wonder of realization that Q-tip fits perfectly inside my ear

the caress of cotton gives way to the splinter of its wooden shaft

can’t I push it in further?

no, no, no, don’t touch

fat, flat wooden sticks

like popsicles licked clean

I grab a fistful from the jar

no, no, no, don’t touch

my big toe flies up

the doctor tickles the bottom of my foot

the reflex hammer looks like an angular lollipop

no, no, no, don’t touch

my pot belly has ballooned from inhaling pints of ice cream

my bald spot made bigger because I keep plucking hairs

nailbeds worried open from pulling at hangnails

no, no, no, don’t touch

FTD C9ORF

spell that in my alphabet cereal

Mother grasps the diagnosis given to me, her husband

no, no, no, don’t touch

the child is father of the man

I grasp her hands

*So was it when my life began;*

*So is it now I am a man;*

*So be it when I shall grow old,*

* Or let me die!*

*The Child is father of the Man*

*–William Wordsworth, “My Heart Leaps Up”*

